# Whey protein sweetened with *Stevia rebaudiana* Bertoni (Bert.) increases mitochondrial biogenesis markers in the skeletal muscle of resistance-trained rats

**DOI:** 10.1186/s12986-019-0391-2

**Published:** 2019-09-13

**Authors:** Yago Carvalho Lima, Mirian Ayumi Kurauti, Gabriel da Fonseca Alves, Jonathan Ferezini, Silvano Piovan, Ananda Malta, Fernanda Losi Alves de Almeida, Rodrigo Mello Gomes, Paulo Cezar de Freitas Mathias, Paula Gimenez Milani, Silvio Cláudio da Costa, Cecilia Edna Mareze-Costa

**Affiliations:** 1 Department of Physiological Sciences, Universidade Estadual de Maringá(UEM), Av. Colombo 5790, Zona 7, Bloco H79, Maringá, PR 87020900 Brazil; 2 Department of Cell Biology and Genetics, Universidade Estadual de Maringá, Maringá, PR Brazil; 3 Department of Morphological Sciences, Universidade Estadual de Maringá, Maringá, PR Brazil; 4 Department of Physiological Sciences, Universidade Federal de Goiás, Goiânia, GO Brazil; 5 Department of Biochemistry, Universidade Estadual de Maringá, Maringá, PR Brazil

**Keywords:** Adipocyte diameter, Mitochondrial function, Muscle fiber diameter, Natural sweeteners, Strength training

## Abstract

**Background:**

A combination of resistance training and whey protein supplementation is a common practice among athletes and recreational exercisers to enhance muscle growth and strength. Although their safety as food additives is controversial, artificial sweeteners are present in whey protein supplements. Thus, natural sweeteners extracted from the leaves of *Stevia rebaudiana* are a potential alternative, due to their safety and health benefits. Here, we investigated the effects of whey protein sweetened with *S. rebaudiana* on physical performance and mitochondrial biogenesis markers in the skeletal muscle of resistance-trained rats.

**Methods:**

Forty male Wistar rats were distributed into four groups: sedentary rats, trained rats, trained rats receiving whey protein and trained rats receiving whey protein sweetened with *S. rebaudiana* leaf extracts. Resistance training was performed by climbing a ladder 5 days per week, during 8-weeks. The training sessions consisted of four climbs carrying a load of 50, 75, 90, and 100% of the maximum load-carrying capacity which we determined before by performing a maximum load-carrying test for each animal. After this period, we collected plasma and tissues samples to evaluate biochemical, histological and molecular (western blot) parameters in these rats.

**Results:**

Dietary supplementation with whey protein sweetened with *S. rebaudiana* significantly enhanced the maximum load-carrying capacity of resistance-trained rats, compared with non-sweetened whey protein supplementation. This enhanced physical performance was accompanied by an increase in the weight of the gastrocnemius and soleus muscle pads. Although the muscle pad of the biceps brachii was not altered, we observed a significant increase in PGC-1α expression, which was followed by a similar pattern in TFAM protein expression, two important mitochondrial biogenesis markers. In addition, a higher level of AMPK phosphorylation was observed in these resistance-trained rats. Finally, supplementation with whey protein sweetened with *S. rebaudiana* also induced a significant decrease in retroperitoneal adipocyte diameter and an increase in the weight of brown adipose tissue pads in resistance-trained rats.

**Conclusion:**

The addition of *Stevia rebaudiana* leaf extracts to whey protein appears to be a potential strategy for those who want to increase muscular mass and strength and also improve mitochondrial function. This strategy may be useful for both athletes and patients with metabolic disorders, such as obesity and type 2 diabetes.

## Background

The use of dietary supplements to improve physical performance is a common practice in athletes and recreational exercisers. Currently, protein supplementation is the most common type of dietary supplementation and is used in combination with resistance exercise training [1, 2]. This type of exercise is very efficient at increasing muscle mass and strength [3, 4], and concomitant protein supplementation can enhance these effects [5, 6].

Dietary protein supplements derived from whey are the most common types of supplements. Whey is the liquid resulting from milk coagulation that occurs during the manufacturing of cheese and other dairy products. Apart from water, lactose, protein, vitamins, and minerals are the main components of whey and these are concentrated to produce dietary protein supplements [7]. Commercial whey protein supplements are classified according to their protein content, varying between 29 and 89% in the whey protein concentrate and ≥ 90% in the isolate [8]. Although the whey protein isolate contains the highest protein concentration, the concentrate has more bioactive components and proteins, making it very attractive for use as a supplement [9].

After resistance exercise, an increase in protein synthesis is observed in skeletal muscles; however, the higher rate of protein breakdown maintains a catabolic state until adequate nutrients are available for recovery. Whey protein is a source of relevant amino acids, including branched-chain amino acids (BCAAs), specially leucine, which is important to the translation initiation of protein synthesis [8]. Therefore, post-exercise supplementation with whey protein can be an effective strategy to improve the recovery of the protein synthesis after resistance exercise, enhancing muscular growth and strength.

The majority of whey protein supplements are artificially sweetened with sucralose and acesulfame potassium. Although the use of these sweeteners is widespread, their safety as food additives remain controversial [10, 11]. There is no evidence about nutritional benefits and as reported by Suez and colleagues [12], artificial sweeteners induced glucose intolerance through alterations of compositional and functional to the gut microbiota, showing a deleterious effects on glucose homeostasis. Thus, there is great interest in the food industry to develop natural sweeteners with potential functional properties to replace those former sweeteners.

There are several sources of natural sweeteners, but the plant *Stevia rebaudiana* Bertoni (Bert.) deserves attention. This plant has been used for centuries by Guarani tribes from Paraguay and Brazil to sweeten herbal infusions and medicinal teas [13]. Several compounds have been extracted from the leaves of *S. rebaudiana*, including steviol glycosides, which are characterized by a sweet taste. Stevioside is the most abundant steviol glycoside found in the leaves of *S. rebaudiana*, followed by rebaudioside A [13, 14], which has a higher sweetness potency than stevioside [15].

Nutrients and other bioactive compounds are also found in the leaves of *S. rebaudiana* and these may be responsible for the well-known functions of this plant, such as antimicrobial and antioxidant activities [13, 16]. Furthermore, several health-promoting properties have been associated with the compounds present in the leaves of *S. rebaudiana*. For example, antidiabetic, anticarcinogenic, antihypertensive, and anti-inflammatory activities have all been associated with the consumption of the leaf extracts of this plant [17]. Thus, we hypothesized that leaf extracts of *S. rebaudiana* may be a potential alternative to artificial sweeteners to enrich the functional properties of whey protein supplements and improve their effects on physical performance and health.

## Methods

### Animals

Forty male Wistar rats (50-days-old) from the Universidade Estadual de Maringá (UEM), were randomly assigned to four equal sized groups: sedentary control rats (SC), trained control rats (TC), trained rats receiving whey protein (TW), and trained rats receiving whey protein sweetened with *S. rebaudiana* leaf extracts (TWS). These rats were housed collectively (5 animals per cage) and weighed once per week, before (week 0) and during the resistance training program (week 1–8). The food intake was also measured once per week, before (at the first day of the week 1) and during the resistance training program. All animals were maintained on a 12 h light/dark cycle, with a controlled temperature and were allowed to freely feed (Nuvilab CR-1, Nuvital Nutrientes S.A., Colombo, PR, Brazil) and drink tap water.

### Resistance training protocol and maximum load-carrying test

Resistance training was performed according to the animal model described by Horberger and Farrar [18], which mimics progressive resistance exercises in humans. There were some minor modifications in this described method, which consisted in the length of the ladder, the dimension of the house chamber at the top of the ladder and the number of training sessions per week. Firstly, trained rats (TC, TW, and TWS groups) were familiarized with climbing a ladder (105 × 5 cm, 1 cm grid, and 80° incline) and with a load apparatus secured to the proximal portion of the tail. At the top of the ladder, the rats reached a house chamber (9 × 9 × 9 cm), where they were allowed to rest between climbs. To familiarize the rats with the exercise protocol, they were kept in the house chamber for 2 min before each attempt to climb. Three attempts were performed per day and the rats were encouraged to climb 20, 50, and 80 cm until they reached the house chamber. Rats were encouraged to climb the ladder by touching their tails. This protocol was performed over three consecutive days. After this period, we determined the maximum load-carrying capacity of each animal at the first day of the week 1 (on Monday), before the beginning of the resistance training program. The test for load capacity was initiated with a climb carrying a load of 75% of the rat’s body weight and upon successful completion, an incremental load of 10% of rat’s body weight was added to the load apparatus. This procedure was repeated until the rats failed to climb steadily. Failure was defined as the inability to climb the ladder following three successive tail stimuli. The highest load successfully carried was considered the maximum load-carrying capacity of the rat. Subsequent resistance training sessions consisted of four climbs carrying a load of 50, 75, 90, and 100% of the previous maximum load. The length of the ladder required the animals to make 8–12 dynamic movements (reps) per climb. This resistance training was performed 5 days per week with 2 days of rest, during an 8-week period. During this period, maximum load-carrying capacity was determined once a week (on Mondays) to set the appropriate load for each animal.

### Whey protein concentrate

Whey was provided by the dairy manufacturer, Flora Milk (Flórida, PR, Brazil) and processed at NEPRON (Núcleo de Estudos em Produtos Naturais, UEM, Maringá, PR, Brazil), as in a previous study [19]. To obtain a whey protein concentrate, samples were processed by ultrafiltration, diafiltration, nanofiltration, and spray drying. The ultrafiltration and diafiltration processes were performed using two polyethersulfone membrane filters with a 10 kDa cut-off (Koch Membrane Systems Inc., Wilmington, MA, USA) in a spiral configuration, with a 50 cm^2^ area. The nanofiltration process was performed using a reverse osmosis system composed of two polyamide membranes with cut-off molecular masses of 180 Da (Koch Membrane Systems Inc.) and 500 Da (Merck Millipore, Burlington, MA, USA), both in a spiral configuration with a 50 cm^2^ area. The whey protein concentrate was dried in an atomized spray dryer (Buchi B-191; BUCHI Brasil Ltda, Valinhos, SP, Brazil) using an input temperature of 170 °C, an output temperature of 105 °C, and a flow of 8 mL × min^− 1^. The total protein, total lipid, lactose, and fixed mineral residue content of the whey protein concentrate were measured, as shown in Table 1. After these analyses were performed, a portion of the whey protein concentrate was sweetened with the leaf extracts of *S. rebaudiana*, rich in rebaudioside A and phenolic compounds, which was obtained as described below.

**Table 1:** Composition of whey protein concentrate obtained in the NEPRON-UEM

	g × 100 g^− 1^ of whey protein concentrate
Total proteins	74,3 ± 0,03
Total lipids	5,16 ± 0,01
Lactose	17,3 ± 0,05
Fixed mineral residues	0,76 ± 0,01

Data are presented as mean ± SEM

### Extraction of sweetener from *S. rebaudiana*

The seminal variety of *S. rebaudiana*, Stevia UEM-13 [20], cultured at NEPRON-UEM, was collected at the maximum vegetative growth stage and dried in an oven at 60 °C. The leaves were then separated and immersed in ethanol (99.5%) for 24 h in the dark at room temperature, to obtain the first fraction. This same procedure was repeated until seven fractions were obtained. The extracts were then dried in a rotary evaporator (BUCHI Brasil Ltda) at 50 °C under vacuum, as described previously [20]. The concentration of glycosides, phenolic compounds, and total flavonoids and the percentage inhibition of DPPH (2,2-diphenyl-1-picrylhydrazyl) were evaluated in the final extract (Table 2).

**Table 2:** Composition and the percentage inhibition of DPPH of *S. rebaudiana* leaf extracts obtained in the NEPRON-UEM

	g × 100 g^− 1^ of whey protein concentrate
Glycosides	26,0 ± 0,01
Phenolic compounds	7,27 ± 0,01
Total Flavonoids	2,80 ± 0,01
DPPH^a^ inhibition	78% × mg^− 1^

^a^DPPH, 2,2-diphenyl-1-picrylhydrazyl. Data are presented as mean ± SEM

### Whey protein supplementation

At the end of exercise training sessions, trained rats from the TW and TWS groups received 100 mg × kg^− 1^ body weight of whey protein, pure (TW) or sweetened with 0.2% *S. rebaudiana* extract (TWS), dissolved in water, by gavage. Control rats from the SC and TC groups underwent the same experimental procedures, but received only water.

### Plasma and tissues sample collection

Forty-eight hours after the last exercise training session, rats underwent a 12 h fast and were then anesthetized with intraperitoneal (ip) administration of 40 mg × kg^− 1^ sodium pentobarbital. Blood samples were collected from the inferior vena cava and placed into tubes containing the anticoagulant, heparin. Plasma samples were obtained by centrifugation (1000 g for 15 min at 4 °C) and were stored at − 80 °C for subsequent biochemical analyses. Rats were then killed by anesthetic overload (120 mg × kg^− 1^ sodium pentobarbital). Skeletal muscles (gastrocnemius, soleus, and biceps brachii), white adipose tissue (WAT; retroperitoneal, perigonadal, and subcutaneous), and brown adipose tissue (BAT) were dissected and weighed. Biceps muscle samples were snap-frozen in liquid nitrogen and stored at − 80 °C for subsequent analysis by western blotting.

### Biochemical analyses

Plasma glucose, triglycerides, total cholesterol, and high-density lipoprotein concentrations were determined using colorimetric methods (Gold Analisa, Belo Horizonte, Brazil) and spectrophotometry (Bioplus2000®; Bioclin, Sao Paulo, Brazil). The 2,2-azinobis-3-ethyl-benzotiazolin-6-sulfonic acid radical was used to analyze total antioxidant capacity (CAT) in blood samples [21]. Plasma insulin concentration was measured by radioimmunoassay [22].

### Muscle fiber diameter measurements

Fragments from medial third of skeletal muscles (gastrocnemius, soleus and biceps brachii) were snap-frozen in liquid nitrogen and stored at − 80 °C. Semi-serial 10 μm-thick cross-sections were stained with hematoxylin and eosin. Skeletal muscle section images were captured at 20× magnification with a BX 50 microscope (Olympus, Tokyo, Japan) and the minor diameter of 100 muscle fibers (per animal) were analyzed using Image-Pro® Plus software version 4.5 (Media Cybernetics Inc., Rockville, MD, USA).

### Adipocyte diameter measurements

Adipocytes were isolated according to Rodbell [23], with minor modifications as previously described [24]. Retroperitoneal fat pads were removed, fragmented with scissors, and incubated in 4 mL of Dulbecco’s Modified Eagle’s Medium containing 25 mmol × L^− 1^ HEPES, 4% (wt. × vol.^− 1^) bovine serum albumin (BSA) fraction V, and 1.25 mg × mL^− 1^ collagenase type II (pH 7.4 at 37 °C), for 20 min with constant shaking. The digested tissue was then filtered and washed 3 times with 25 mL of Earle’s solution containing 20 mmol × L^− 1^ HEPES, 1% (wt. × vol.^− 1^) BSA and 1 mmol × L^− 1^ sodium pyruvate (pH 7.4 at 37 °C). Finally, images of isolated adipocytes (100 cells per animal) were captured at 4× magnification using a BX 50 microscope and adipocyte diameter was measured using Image-Pro® Plus software version 4.5.

### Western blotting

Western blotting analysis was performed as previously described [25], with minor modifications. Samples from the biceps brachii muscle were homogenized in lysis buffer and centrifuged (12,000 g for 20 min at 4 °C) to obtain a protein extract. Total protein concentration was measured and 30 μg protein samples were separated by 12% sodium dodecyl sulfate polyacrylamide gel electrophoresis. Proteins were then transferred onto nitrocellulose membranes and the membranes were blocked with Tris-buffered saline containing 5% (wt. × vol.^− 1^) BSA for 1 h at room temperature. After blocking, membranes were incubated overnight at 4 °C with anti-PGC1α (#PA5–72948; Thermo Fisher, Waltham, MA, USA), anti-TFAM (sc-23,588; Santa Cruz Biotechnology, Dallas, TX, USA), anti-phospho-AMPKα (#2535; Cell Signaling, Danvers, MA,USA), or anti-α-tubulin (T5168; Sigma Aldrich, St Louis, MO, USA) primary antibodies. Bands were detected by chemiluminescence (SuperSignal™ West Fento; Pierce Biotechnology Inc., Rockford, IL, USA) after incubation with an appropriate horseradish peroxidase-conjugated secondary antibody and visualized using the C-DiGit® Blot Scanner (LI-COR Biosciences, Lincoln, NE, USA). Finally, their intensities were analyzed using ImageJ software (National Institutes of Health, Bethesda, MD, USA).

### Statistics

Data were assessed for deviations from a normal distribution. Student’s t-test was used for the statistical analysis of data from two groups (SC vs TC), to assess the effect of the resistance training alone. One-way ANOVA with an unpaired Tukey’s post-hoc test was used to analyze data from three groups (TC vs TW vs TWS), to assess the effect of whey protein supplementation in trained rats, with/without *Stevia rebaudiana* extract. Statistical analyses were performed using Prism software version 5.00 for Windows (GraphPad Software, La Jolla, CA, USA). Significant outliers were identified using Grubbs’ test and removed from these statistical analyses as appropriate. The sample size (n) used for the statistical analysis of each group in the experiments is described in the figure legends. The n for the adipocytes diameter and western blot analysis was smaller due to the limitation of the materials supply and availability of equipment, required to these specific experiments. All data are presented as the mean ± standard error of the mean (SEM) and were considered significantly different if *p* ≤ 0.05.

## Results

### Resistance training induced exercise adaptation in rats (SC vs TC)

**Fig. 1:**
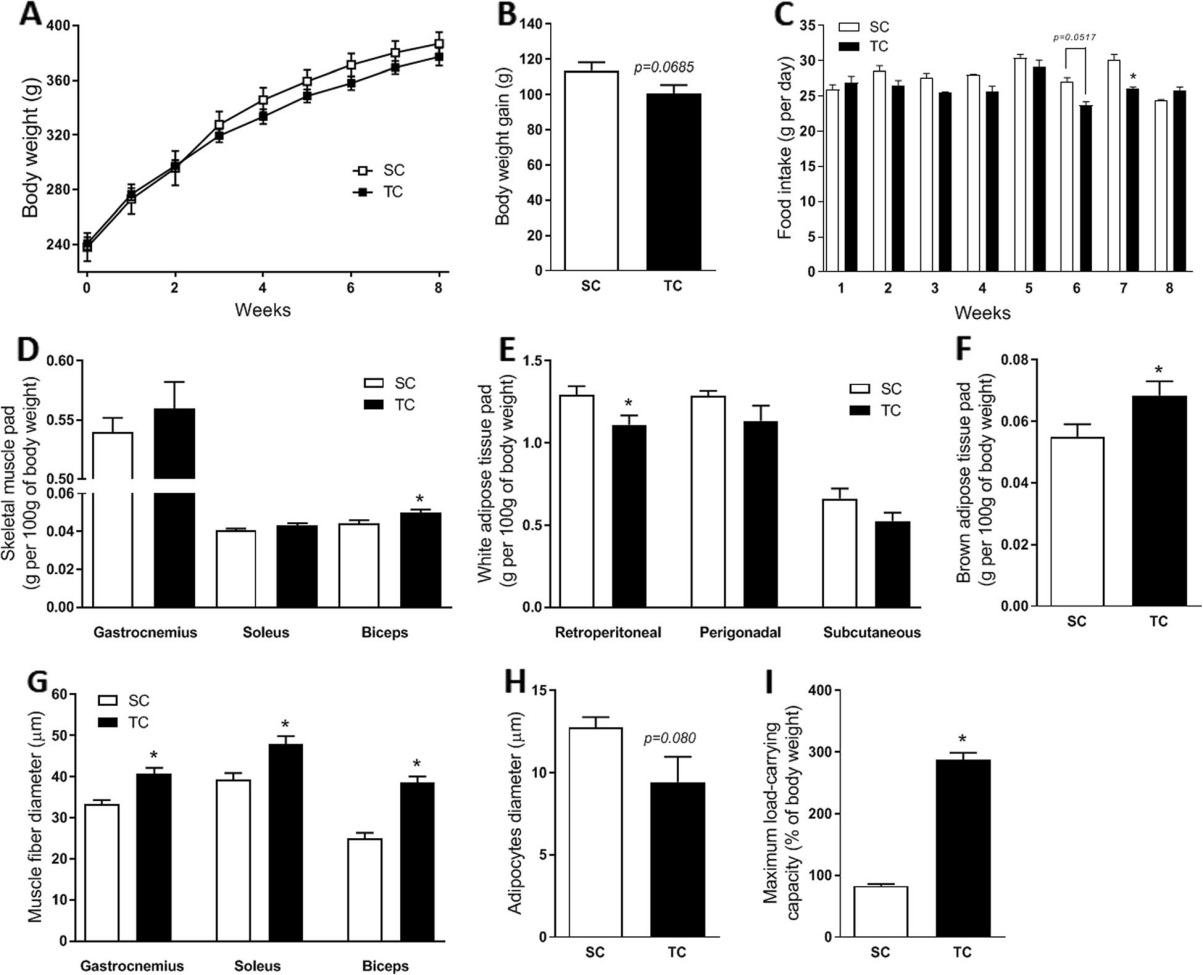
Exercise adaptations in resistance-trained rats. Body weight during the 8-weeks of the resistance training (**a**) and the body weight gain after this period (**b**), *n* = 9 SC and 10 TC. Food intake during the 8-weeks of the resistance training (**c**), *n* = 10. Weight of the skeletal muscles pads (**d**), white adipose tissues pads (**e**) and brown adipose tissue pad (**f**), *n* = 10 SC and 9 TC. Muscle fiber (**g**) and retroperitoneal adipocytes (**h**) diameters, *n* = 6. Maximum load-carrying capacity at the end of resistance training program (I), *n* = 10. For the representative images of muscle fibers and adipocytes diameters, see Additional file 1: Figures S1 and S2. SC, sedentary control rats; TC, trained control rats. Data are presented as the mean ± S.E.M. *p ≤ 0.05 vs SC, Student t-test

First of all, to assess the effect of the resistance training alone, we compared the TC group with the SC group. As expected, after 8 weeks of resistance training, the maximum load carried by TC rats was higher, compared with the maximum load carried by SC rats (Fig. 1i). Moreover, resistance training seemed to reduce body weight gain (Fig. 1a and b), although this difference was not statistically significant. At the 7th week of training, the food intake of TC group was significantly lower compared with the food intake of SC group (Fig. 1c). The weight of the retroperitoneal WAT pad was also lower in the TC group (Fig. 1e), but the smaller diameter of its adipocyte was not statistically significant (Fig. 1h); however, the BAT pad (Fig. 1f) in TC rats was significantly higher, compared with SC rats. Although resistance training only increased the weight of the biceps brachii muscle pad (Fig. 1d), the fiber diameters of all skeletal muscles evaluated significantly increased with resistance training (Fig. 1g). Among the biochemical parameters analyzed, the only significant difference observed was the higher plasma cholesterol concentration in the TC group, compared with the SC group (Table 3).

**Table 3:** Plasmatic parameters of the sedentary and resistance-trained rats

Parameters	SC	TC	TW	TWS
Glycemia (mg × dl^−1^)	79.2 ± 3.0	82.4 ± 1.9	80.0 ± 1.4	85.0 ± 2.4
Insulinemia (ng × ml^− 1^)	0.33 ± 0.02	0.29 ± 0.05	0.21 ± 0.02	0.33 ± 0.03^&^
Triglyceride (mg × dl^− 1^)	49.6 ± 5.8	50.2 ± 5.3	46.7 ± 4.8	49.6 ± 2.9
Cholesterol (mg × dl^− 1^)	85.1 ± 2.4	108.2 ± 3.1*	106.6 ± 1.7	110.8 ± 2.3
HDL^a^ (mg × dl^− 1^)	36.4 ± 2.7	40.5 ± 1.4	39.3 ± 0.9	38.4 ± 1.9
Total proteins (mg × dl^− 1^)	4.47 ± 0.08	4.41 ± 0.09	4.38 ± 0.04	4.51 ± 0.07
Creatinine (mg × dl^− 1^)	5.33 ± 0.31	5.44 ± 0.29	5.06 ± 0.23	4.85 ± 0.39
TAC^b^ (μmol Trolox equivalent × l^−1^)	0.92 ± 0.15	1.04 ± 0.14	0.80 ± 0.07	0.85 ± 0.10

^a^HDL, high density lipoprotein; ^b^TAC, total antioxidant capacity. Data are presented as mean ± SEM, *n* = 8–10. *p ≤ 0.05 vs SC, Student t-test. ^#^*p* ≤ 0.05 vs TC and ^&^*p* ≤ 0.05 vs TW, ANOVA, Tukey’s post-hoc test

### Whey protein sweetened with *S. rebaudiana* increased the maximum load-carrying capacity in resistance-trained rats (TC vs TW vs TWS)

**Fig. 2:**
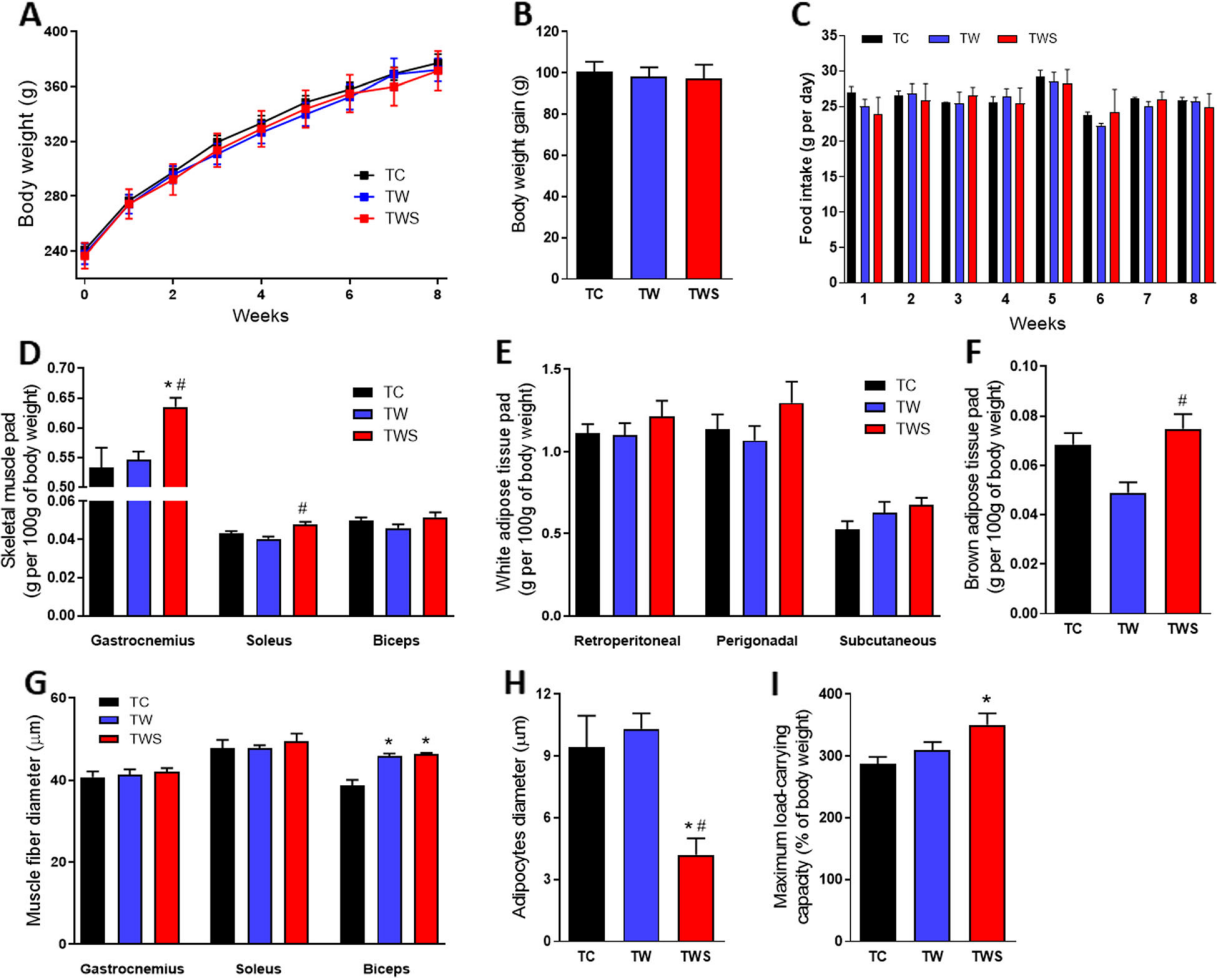
Effect of the supplementation with whey protein sweetened with *S. rebaudiana* on the exercise adaptations in resistance-trained rats. Body weight during the 8-weeks of the resistance training (**a**) and the body weight gain after this period (**b**), *n* = 10. Food intake during the 8-weeks of the resistance training (**c**), *n* = 10. Weight of the skeletal muscles pads (**d**), white adipose tissues pads (**e**) and brown adipose tissue pad (**f**), *n* = 9 TC, 9 TW and 10 TWS. Muscle fiber (**g**) and retroperitoneal adipocytes (**h**) diameters, *n* = 6. Maximum load-carrying capacity at the end of resistance training program (**i**), *n* = 10. For the representative images of muscle fibers and adipocytes diameters, see Additional file 1: Figures S1 and S2. TC, trained control rats; TW, trained rats receiving whey protein; TWS, trained rats receiving whey protein sweetened with *S. rebaudiana* leaf extracts. Data are presented as the mean ± S.E.M. *p ≤ 0.05 vs TC and ^#^*p* ≤ 0.05 vs TW, ANOVA, Tukey’s post-hoc test

**Fig. 3:**
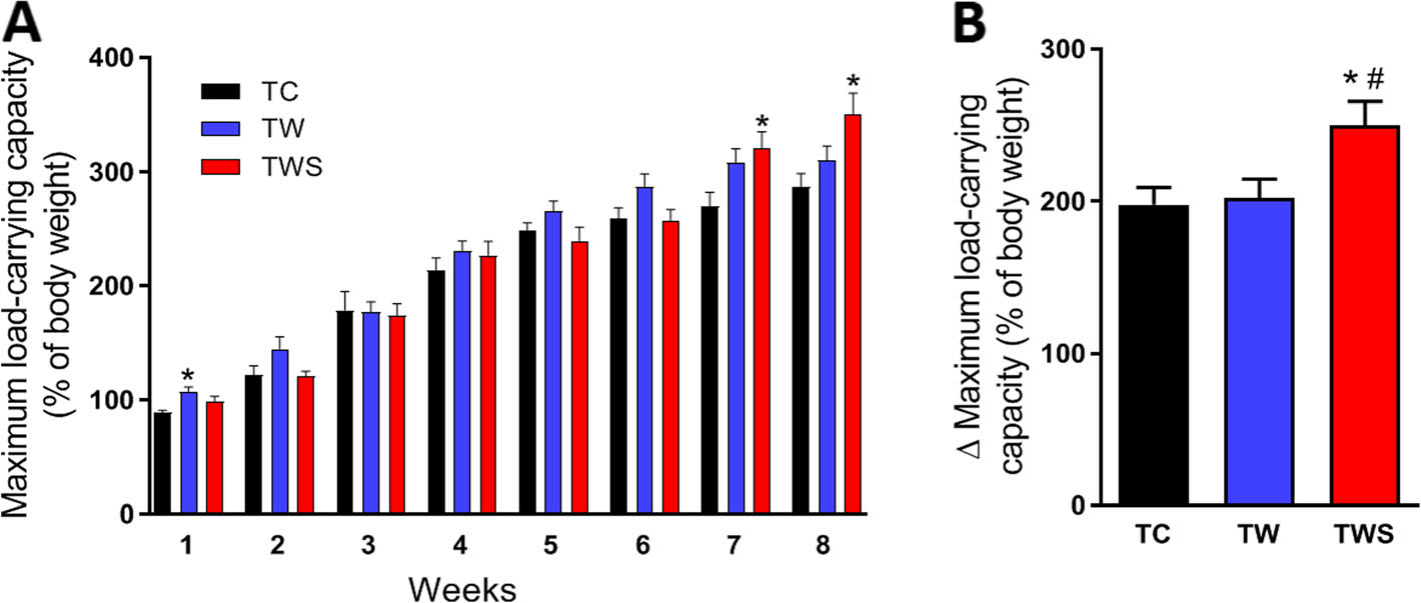
Effect of the supplementation with whey protein sweetened with *S. rebaudiana* on the incremental increase of the maximum load-carrying capacity in resistance-trained rats. Maximum load-carrying capacity (**a**) and its incremental increase (**b**) during the 8-weeks of the resistance training program. TC, trained control rats; TW, trained rats receiving whey protein; TWS, trained rats receiving whey protein sweetened with *S. rebaudiana* leaf extracts. *n* = 10. Data are presented as the mean ± S.E.M. *p ≤ 0.05 vs TC and ^#^*p* ≤ 0.05 vs TW, ANOVA, Tukey’s post-hoc test

To assess the effect of whey protein supplementation in trained rats, with/without *Stevia rebaudiana* extract, we analyzed the differences between the TC, TW and TWS groups. Supplementation with whey protein alone did not significantly increase maximum load-carrying capacity in rats after 8 weeks of resistance training, as shown in Fig. 2i. No differences in body weight, food intake, skeletal muscle, or WAT and BAT pads were observed in the TW group compared with the TC group (Fig. 2a–f). Although supplementation with whey protein did not change the skeletal muscles pads, muscle fiber diameter of the biceps brachii was higher in the TW group compared with the TC group (Fig. 2g). Interestingly, supplementation with whey protein sweetened with the *S. rebaudiana* leaf extracts, significantly increased maximum load-carrying capacity in rats after 8 weeks of resistance training (Fig. 3b) and this alteration was evident from the 7th to 8th week of training (Fig. 3a). Thus, the difference between the initial and final maximum load-carrying capacity was greater in the TWS group than in the TC and TW groups (Fig. 3b). After 8 weeks of training, the weight of the gastrocnemius muscle pad was higher in TWS rats compared to both TC and TW rats, but there were no differences in the diameter of these muscle fibers between the groups (Fig. 2d and g). Similar to those data observed in the TW rats, the weight of the biceps brachii muscle of the TWS rats was not different, but the diameter of its fibers was higher compared to the diameter of the respective fibers in TC rats (Fig. 2d and g). Although no difference were seen in the WAT pads of TWS rats, the diameter of the retroperitoneal adipocytes was lower in these rats compared with the TC and TW rats (Fig. 2e, f, and h). Moreover, the weight of the BAT pad observed in the TWS group, was significantly higher, compared to the TW group (Fig. 2f).

### Whey protein sweetened with *S. rebaudiana* increased plasma insulin concentration in resistance-trained rats

Whey protein supplementation has been known to increase the release of insulin into the blood circulation, but we did not observe this effect in the TW group compared to the TC group (Table 3). However, the plasma insulin concentration in TWS rats was significantly higher, compared with the TW rats (Table 3).

### Whey protein sweetened with *S. rebaudiana* increased mitochondrial biogenesis markers in the skeletal muscle of resistance-trained rats

**Fig. 4:**
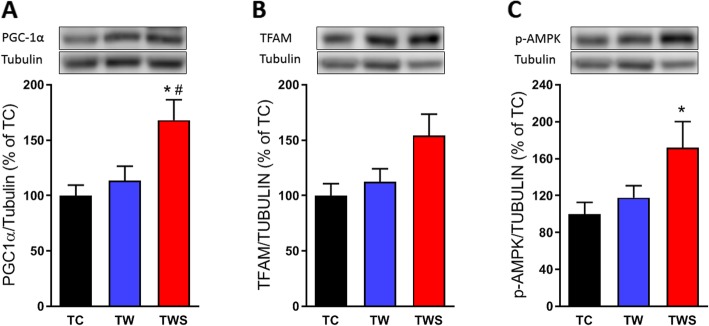
Effect of the supplementation with whey protein sweetened with *S. rebaudiana* on the protein expression of mitochondrial biogenesis markers in resistance-trained rats. Protein expression of PGC-1α (**a**), TFAM (**b**) and phospho-AMPKα^Thr172^ (**c**) in the biceps brachii muscle of the resistance-trained rats and their representatives immunoblotting images. TC, trained control rats; TW, trained rats receiving whey protein; TWS, trained rats receiving whey protein sweetened with *S. rebaudiana* leaf extracts. *n* = 5 TC, 6 TW and 6 TWS. Data are presented as the mean ± S.E.M. *p ≤ 0.05 vs TC and ^#^*p* ≤ 0.05 vs TW, ANOVA, Tukey’s post-hoc test

Considering the similar biceps brachii fiber diameter between TW and TWS rats (Fig. 2g), we asked whether a molecular change would be observed between these groups. To answer this question, we investigated the expression of an important mitochondrial biogenesis marker, peroxisome proliferator-activated receptor γ coactivator 1-α (PGC-1α), in this muscle. Interestingly, the expression of this protein was significantly higher in the biceps of TWS rats, compared with the TC and TW rats (Fig. 4a). In addition, we evaluated mitochondrial transcriptional factor A (TFAM or mtTFA), a protein downstream of PGC-1α in the mitochondrial biogenesis regulation pathway. Although it was not statistically significant, the expression of this protein accompanied the pattern of PGC-1α expression in the biceps brachii of TWS rats, as evidenced in Fig. 4b. Finally, we also observed a higher level of phosphorylation of AMP-activated protein kinase (AMPK), a protein upstream of PGC-1α, in the biceps brachii of TWS rats compared with the TC rats (Fig. 4c).

## Discussion

A combination of resistance training and whey protein supplementation is a common practice of athletes and recreational exercisers to enhance muscle growth and strength. The majority of commercial whey protein supplements are artificially sweetened; however, the safety of these sweeteners as food additives remains unclear [10]. Therefore, natural sweeteners, such as those extracted from the leaves of *S. rebaudiana*, offer an interesting alternative, due to the known safety and health benefits that they provide [17, 26]. Here, we demonstrated a significant increase in the maximum load-carrying capacity of resistance-trained rats administered a whey protein supplement sweetened with *S. rebaudiana*. This improvement in physical performance was accompanied by a higher size of the gastrocnemius and soleus muscles pads. Although the biceps brachii muscle pad was not affected, we observed an interesting effect on mitochondrial biogenesis markers in this muscle. The PGC-1α expression was significantly higher in resistance-trained rats administered with a whey protein supplement sweetened with *S. rebaudiana*, and this result was followed by a similar pattern in TFAM expression in these same rats. These effects were associated with a higher level of AMPK phosphorylation. Therefore, the addition of *S. rebaudiana* leaf extracts to whey protein is a potential approach for improving the ability of this supplement to promote greater physical performance in resistance-trained subjects.

Resistance training, also known as strength training, is a well-established strategy to increase muscle mass and strength. Despite resulting in a higher level of protein breakdown, resistance exercise has been shown to increase protein synthesis in skeletal muscle [27, 28], which may explain the great effect of this type of exercise on muscle growth. Here, we demonstrated a higher muscle fiber diameter (Fig. 1g) and a greater maximum load-carrying capacity (Fig. 1i) in rats after resistance training. In addition, we observed a slight reduction in body weight gain (Fig. 1b) in these rats, which is not unexpected, considering that they expended more energy and displayed lower food intake (Fig. 1c) than sedentary rats. These factors have been associated with the ability of resistance exercise to lower ghrelin concentration in the circulation [29].

The balance between protein synthesis and breakdown depends on the availability of nutrients, primarily amino acids. Thus, protein supplements intake after resistance exercise can improve the recovery of protein synthesis, thus favoring muscular growth [5]. Whey protein is an ideal supplement to achieve this purpose, since it is rapidly digested and absorbed in the gastrointestinal tract and it provides several amino acids to enable protein synthesis. In addition, whey protein is an important source of BCAAs, such as leucine [8], which is a critical regulator of translation initiation [30]. Thus, whey protein supplementation can greatly enhance muscular growth and strength with a concomitant resistance training program. In the present study, we observed a greater increase in fiber diameter in the biceps brachii muscle of resistance-trained rats that ingested whey protein immediately after each exercise session (Fig. 2g). Studies in resistance-trained elderly humans have shown that the timing of post-exercise protein intake can affect muscle hypertrophy, with greater effects being observed if post-exercise protein intake occurs soon after exercise [31].

Natural sweeteners, especially glycosides derived from the leaves of *S. rebaudiana* Bertoni (Bert.), have been associated with several health benefits [17]. Previously, we demonstrated that whey protein fortified with a fraction (free of steviol glycosides) extracted from *S. rebaudiana* significantly increases the antioxidant activity of this supplement and improves glycemic control in diabetics rats [16]. Thus, we hypothesized that adding an extract (with steviol glycosides) from the leaves of *S. rebaudiana* to whey protein, would not only sweeten the taste, but may also improve the physical performance-enhancing effects of the supplement. As expected, supplementation with whey protein sweetened with *S. rebaudiana*, enhanced the maximum load-carrying capacity of resistance-trained rats, compared to rats supplemented with non-sweetened whey protein (Fig. 3). This improvement was accompanied by a higher size of the gastrocnemius and soleus muscle pads; however, no effects on muscle fiber diameter were observed in these muscles (2D and 2G). The larger gastrocnemius and soleus muscle pads may explain, at least in part, the enhanced load-carrying capacity of these rats. It is possible that the insulinotropic effects of *S. rebaudiana* [32] enhanced the whey protein-induced protein synthesis in these skeletal muscles, as suggested by the higher levels of insulinemia found in those rats (Table 3). However, further studies are necessary to confirm this hypothesis.

The antioxidant activity of *S. rebaudiana* is well established [13, 17]; however, the effect of these antioxidant compounds in association with resistance exercise is unclear. It is possible that the combination of these antioxidant compounds and resistance training, may cause detrimental effects on the adaptations to this type of exercise [33, 34]. Thus, the improvement in physical performance, induced by the addition of *S. rebaudiana* to the whey protein supplement, maybe due to other mechanisms than its antioxidant activity. Recent studies have shown that compounds extracted from the leaves of *S. rebaudiana* stimulate the activation of AMPK, which has an important role during exercise [35, 36]. However, this protein seems to be preferentially activated by endurance, rather than resistance, exercise [37]. Although the activation of AMPK by resistance exercise remains unclear [38, 39], we observed a lower phosphorylation levels of this protein in the biceps brachii muscle of resistance-trained rats (Additional file 1: Figure S3). Interestingly, supplementation with whey protein sweetened with *S. rebaudiana* increased the phosphorylation of AMPK (Fig. 4c) in this muscle of resistance-trained rats, suggesting that some endurance exercise adaptations may be also enhanced. An increase in the number and function of mitochondria are well-known adaptations related to endurance exercise, which has been shown to induce mitochondrial biogenesis [40]. Here, we provide evidence that supplementation with whey protein sweetened with *S. rebaudiana*, may induce mitochondrial biogenesis in resistance-trained rats, as indicated by the higher levels of PGC-1α protein expression (Fig. 4a and b), which are an important marker of this processes [41]. The activation of AMPK pathways may be involved in the enhanced mitochondrial biogenesis, as previously reported [42, 43]. Thus, identifying the mechanisms whereby *S. rebaudiana* activates this pathway is a challenge for future studies.

Besides enhanced mitochondrial biogenesis, increased PGC-1α expression is also related to increased oxidative phosphorylation activity [44], which in turn, may increase fatty acid oxidation. Thus, the lower size of WAT pads would be expected in association with a higher level of PGC-1α expression in skeletal muscle. We did not observe any effect on WAT pads, but the diameter of retroperitoneal adipocytes was significantly smaller in TWS rats (Fig. 2h). Since large adipocytes are associated with the release of pro-inflammatory adipokines [45], the reduction in adipocyte size induced by the *S. rebaudiana*-sweetened whey protein supplement in resistance-trained rats, suggests that this supplement may be beneficial for obesity and type 2 diabetes therapies. Likewise, the higher weight of BAT pads observed in these rats demonstrates another interesting effect of this supplement, reinforcing its possible beneficial in the treatment of metabolic disorders [46].

## Conclusion

In summary, this study demonstrated that the addition of an *S. rebaudiana* leaf extract to a whey protein supplement can enhance physical performance in resistance-trained rats, as evidenced by an increase in their maximum load-carrying capacity. The increase in the weight of skeletal muscle pads in these rats may contribute to this effect, but other molecular mechanisms may also be involved, as suggested by the increase in protein expression of mitochondrial biogenesis markers. Therefore, a whey protein supplement sweetened with *S. rebaudiana* may be interesting option for athletes undergoing resistance training, who want to increase muscular mass and strength, but also increase mitochondrial function, which is commonly induced by endurance exercise. Finally, this strategy may also be useful for the treatment of patients with metabolic diseases, such as obesity and type 2 diabetes.

## Supplementary information

**Additional file 1. Figure S1.** Representative images of muscle fiber diameters analysis. **Figure S2.** Representative images of adipocytes diameters analysis. **Figure S3.** Effect of the resistance training on the protein expression of mitochondrial biogenesis markers in skeletal muscle of rats.

## Data Availability

All data supporting the conclusions of this paper are included in this manuscript and Additional file 1. The raw data are available from the corresponding author on reasonable request.
