# Supplementary material for: Whey protein sweetened with Stevia rebaudiana Bertoni (Bert.) increases mitochondrial biogenesis markers in the skeletal muscle of resistance-trained rats
Source: Nutr Metab (Lond). 2019 Sep 13;16:65. doi: 10.1186/s12986-019-0391-2 (PMC6743177; doi:10.1186/s12986-019-0391-2)
Supplement: Supplementary file 1 — Additional file 1. Figure S1. Representative images of muscle fiber diameters analysis. Figure S2. Representative images of adipocytes diameters analysis. Figure S3. Effect of the resistance training on the protein expression of mitochondrial biogenesis markers in skeletal muscle of rats. [file 12986_2019_391_MOESM1_ESM.pdf]

Whey protein sweetened with *Stevia rebaudiana* Bertoni (Bert.) increases mitochondrial biogenesis markers in the skeletal muscle of resistance-trained rats

Running head: Stevia improve whey protein functions

Yago Carvalho Lima[1]*, Mirian Ayumi Kurauti[1], Gabriel da Fonseca Alves[1], Jonathan Ferezini[1], Silvano Piovan[1], Ananda Malta[3], Fernanda Losi Alves de Almeida[5], Rodrigo Mello Gomes[4], Paulo Cezar de Freitas Mathias[3], Paula Gimenez Milani[2], Silvio Cláudio da Costa[2] and Cecilia Edna Mareze da Costa[1]

[1]Department of Physiological Sciences, Universidade Estadual de Maringá, Maringá, PR, Brazil

[2]Department of Biochemistry, Universidade Estadual de Maringá, Maringá, PR, Brazil

[3]Department of Cell Biology and Genetics, Universidade Estadual de Maringá, Maringá, PR, Brazil

[4]Department of Physiological Sciences, Universidade Federal de Goiás, Goiânia, GO, Brazil

[5]Department of Morphological Sciences, Universidade Estadual de Maringá, Maringá, PR, Brazil

*Correspondent author:

Yago Carvalho Lima

Department of Physiological Sciences, Universidade Estadual de Maringá (UEM)

Av. Colombo 5790, Zona 7, Bloco H79, Maringá, PR, Brazil

Zip code 87020900,

Phone: +55 44 99733-7329

E-mail: yago7_lima@hotmail.com

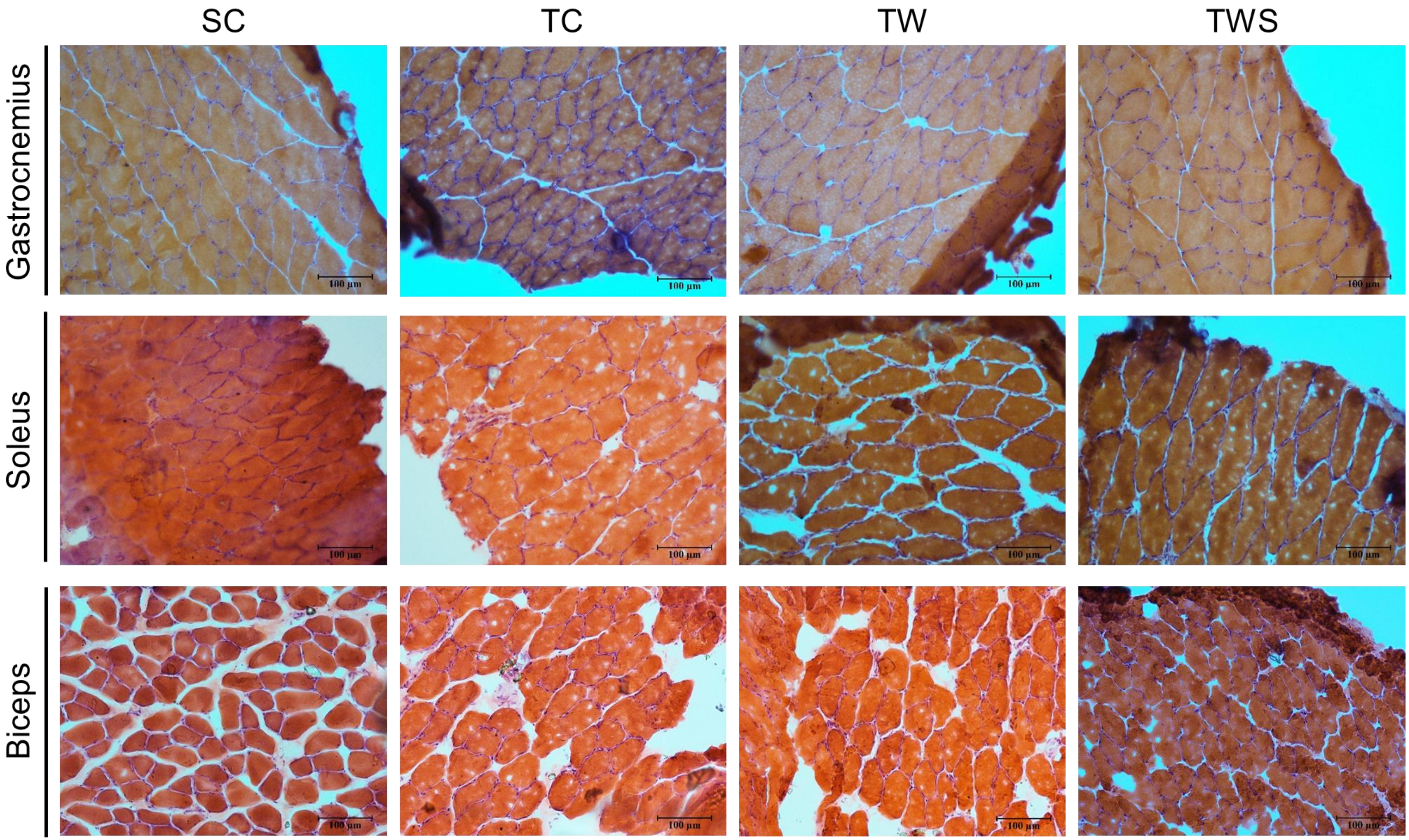

Supplementary Figure 1. Representative images of muscle fiber diameters analysis. Semi-serial cross-sections with 10 µm thick of the skeletal muscles (gastrocnemius, soleus and biceps brachii) were stained with haematoxylin and eosin. The images of these sections were captured at × 20 magnification with a microscope, as described in the Methods. SC, sedentary control rats; TC, trained control rats; TW, trained rats receiving whey protein; TWS, trained rats receiving whey protein sweetened with *S. rebaudiana* leaf extracts.

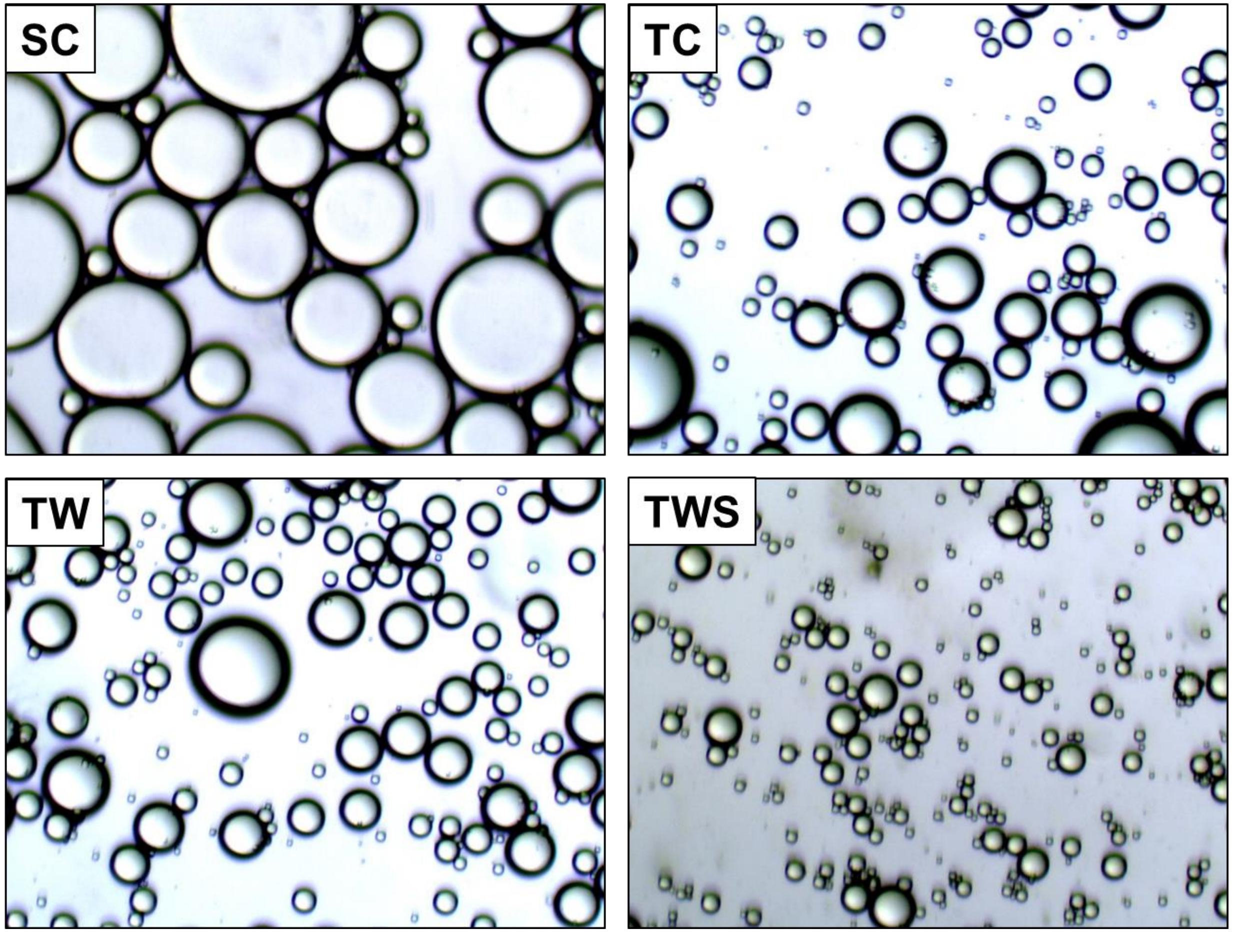

Supplementary Figure 2. Representative images of adipocytes diameters analysis. The adipocytes from retroperitoneal adipose tissue was isolated, as described in the Methods, and the image of these adipocytes were capture at × 4 magnification using a microscope. SC, sedentary control rats; TC, trained control rats; TW, trained rats receiving whey protein; TWS, trained rats receiving whey protein sweetened with *S. rebaudiana* leaf extracts.

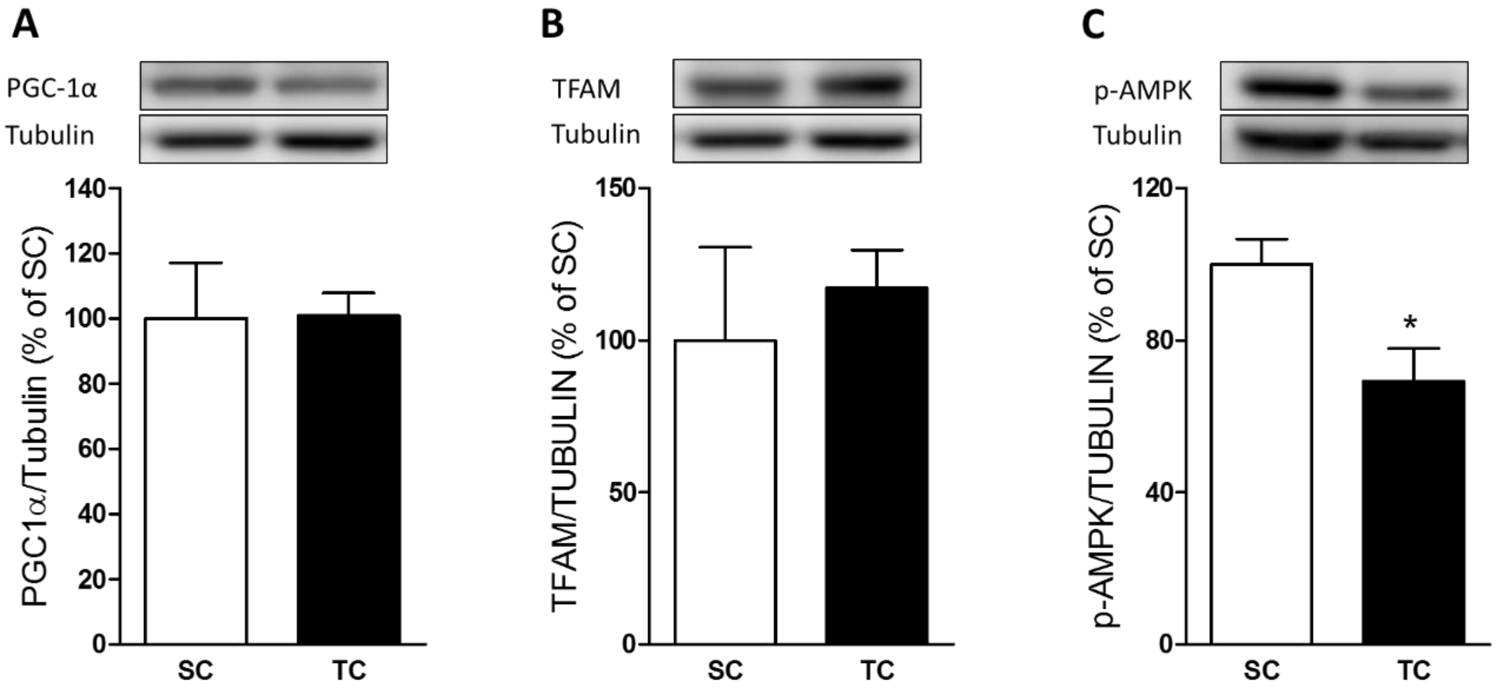

Supplementary Figure 3. Effect of the resistance training on the protein expression of mitochondrial biogenesis markers in skeletal muscle of rats. Protein expression of PGC-1α (A), TFAM (B) and phospho-AMPKα^{Thr172} (C) in the biceps brachii muscle of the resistance-trained rats and their representatives immunoblotting images. SC, sedentary control rats; TC, trained control rats. n = 4-5. Data are presented as the mean ± S.E.M. *$p \leq 0.05$ *vs* SC, Student's t test.
